# The impact of communicating genetic risks of disease on risk-reducing health behaviour: systematic review with meta-analysis

**DOI:** 10.1136/bmj.i1102

**Published:** 2016-03-15

**Authors:** Gareth J Hollands, David P French, Simon J Griffin, A Toby Prevost, Stephen Sutton, Sarah King, Theresa M Marteau

**Affiliations:** 1 Behaviour and Health Research Unit, University of Cambridge, Cambridge, UK; 2 School of Psychological Sciences, University of Manchester, Manchester, UK; 3 Department of Public Health and Primary Care, University of Cambridge, Cambridge, UK; 4 Imperial Clinical Trials Unit, Imperial College London, London, UK

## Abstract

**Objective** To assess the impact of communicating DNA based disease risk estimates on risk-reducing health behaviours and motivation to engage in such behaviours.

**Design** Systematic review with meta-analysis, using Cochrane methods.

**Data sources** Medline, Embase, PsycINFO, CINAHL, and the Cochrane Central Register of Controlled Trials up to 25 February 2015. Backward and forward citation searches were also conducted.

**Study selection** Randomised and quasi-randomised controlled trials involving adults in which one group received personalised DNA based estimates of disease risk for conditions where risk could be reduced by behaviour change. Eligible studies included a measure of risk-reducing behaviour.

**Results** We examined 10 515 abstracts and included 18 studies that reported on seven behavioural outcomes, including smoking cessation (six studies; n=2663), diet (seven studies; n=1784), and physical activity (six studies; n=1704). Meta-analysis revealed no significant effects of communicating DNA based risk estimates on smoking cessation (odds ratio 0.92, 95% confidence interval 0.63 to 1.35, P=0.67), diet (standardised mean difference 0.12, 95% confidence interval −0.00 to 0.24, P=0.05), or physical activity (standardised mean difference −0.03, 95% confidence interval −0.13 to 0.08, P=0.62). There were also no effects on any other behaviours (alcohol use, medication use, sun protection behaviours, and attendance at screening or behavioural support programmes) or on motivation to change behaviour, and no adverse effects, such as depression and anxiety. Subgroup analyses provided no clear evidence that communication of a risk-conferring genotype affected behaviour more than communication of the absence of such a genotype. However, studies were predominantly at high or unclear risk of bias, and evidence was typically of low quality.

**Conclusions** Expectations that communicating DNA based risk estimates changes behaviour is not supported by existing evidence. These results do not support use of genetic testing or the search for risk-conferring gene variants for common complex diseases on the basis that they motivate risk-reducing behaviour.

**Systematic review registration** This is a revised and updated version of a Cochrane review from 2010, adding 11 studies to the seven previously identified.

## Introduction

Searching for gene variants associated with risks of common complex conditions, including diabetes and various cancers, continues to receive considerable attention.^1^ ^2^ Although the main target of such research is more effective treatments, more precise prediction of disease has also been anticipated. Less attention has been given to evaluating whether health benefits, in particular risk-reducing changes in behaviour, can be realised through communicating the results of such predictions. For example, does communicating to smokers that they have an increased genetic risk of developing lung cancer motivate smoking cessation, or does telling middle aged people that they have an increased genetic risk of developing diabetes motivate increased physical activity to reduce this risk? These are particularly timely questions, given high levels of interest in personalised medicine and in direct-to-consumer testing. More than 10 years ago, direct-to-consumer tests for a range of common complex disorders were rushed to market. These tests continue to be sold in Canada, the United Kingdom, and other European countries, including Denmark, Finland, the Netherlands, Sweden, and Ireland (www.23andme.com/en-gb/health/; www.23andme.com/en-eu/), with continued international expansion likely. In the United States, expansion was tempered in 2013 when the Food and Drug Administration ordered the company 23andme to stop selling its testing kits because of concerns about their accuracy and usefulness, but as of October 2015 the company has resumed selling some health related services. Regulatory systems in the USA are now being developed to ensure public protection in anticipation of rapid developments in precision medicine, including increased commercial interests in direct-to-consumer genomic testing.^3^

As the science develops, it is increasingly possible to provide information about multiple single genes, each relating to different disease risks, and also to aggregate multiple risk loci and identify patterns of characteristics across multiple genes that in combination confer increased risks of one or more diseases. However, DNA based disease risk estimates will only translate into health benefits if acting on them modifies disease outcomes, and if those informed of these genetic risks undertake the relevant actions.

Three competing predictions on the effect of communicating DNA based disease risks are evident in the literature. Firstly, communicating DNA based risk estimates, particularly if based on the detection of risk-conferring mutations, motivates behaviour change more strongly than does communicating risks of disease derived from other types of risk information.^4^ ^5^ ^6^ ^7^ This is consistent with theories of attitude change, which suggest that the greater the personal salience of information, such as that regarding one’s own DNA, the greater the impact.^8^ Secondly, communicating DNA based disease risk estimates demotivates behaviour change.^9^ This is based on the observation that diseases considered to have a genetic basis are perceived as less controllable,^10^ and using DNA to estimate disease risks may lead to a sense of fatalism or lack of control over the ability to improve outcomes.^11^ Finally, communicating such information is likely to have, at best, only a small effect on behaviour. This is based on review evidence showing that perceptions of disease risk exert, at most, only a small influence on behaviour,^12^ and that communicating the results of a wide range of biomarker tests has no consistent effect on behaviour.^13^ ^14^

Several narrative reviews have been conducted assessing the emotional and behavioural outcomes of communicating DNA based disease risk estimates^15^ ^16^ ^17^ ^18^ and the outcomes of genetic health services for common adult onset conditions.^19^ However, these reviews identified few clinical studies using randomised designs to assess effects on behaviour and did not include quantitative syntheses of effects. Although systematic reviews have been conducted more recently, these have focused on single behaviours such as smoking cessation.^20^ ^21^ We assessed the impact of communicating DNA based disease risk estimates on risk-reducing behaviours and motivation to undertake such behaviours. We also examined whether communicating the presence of a risk-conferring genotype would elicit a stronger (and potentially counteractive) motivational response than communicating its absence.^22^

There are high expectations that advances in genetics will usher in a new era of personalised medicine, and that because communicating genetic risks will motivate risk-reducing behaviour changes, such communication has a role in risk reduction strategies aimed at improving population health.^23^ The results of this review will inform debates about the role of genetic testing in public health policies. The findings will also contribute to the evidence base on the behavioural impact of communicating risks of disease based on a wide range of biological markers, of which DNA is but one.^13^ ^14^ ^24^

## Methods

This is a revised and updated version of a Cochrane review from 2010,^25^ adding 11 studies to the seven previously identified. The methods are described in detail elsewhere.^25^

### Data sources

We searched Medline, Embase, PsycINFO, CINAHL, and the Cochrane Central Register of Controlled Trials up to 25 February 2015. Backward and forward citation searches were also conducted from included studies. Appendix 1 details the Medline search strategy.

### Inclusion and exclusion criteria

To be eligible, studies had to be randomised controlled trials or quasi-randomised controlled trials (controlled trials using a non-random method of allocation to study arm, such as alternation or by date of birth), have recruited adult populations (≥18 years), and include one group that received personalised DNA based risk estimates for diseases for which behaviour change could reduce risk (including heart disease, cancers, and Alzheimer’s disease). We excluded studies that evaluated the communication of DNA based risk estimates of diseases for which there is no known intervention to reduce that risk, such as Huntington’s disease.

The studies assessed the effects of the intervention relative to the effects of communicating non-DNA based disease risk estimates (assessment based on family history, biological markers of disease, personal characteristics, or a combination thereof) or of communicating no disease risk estimates. Included studies therefore formed three main groups, defined by differences in the intervention and comparison groups: disease risk estimates based on DNA versus non-DNA based disease risk estimates; disease risk estimates based on DNA plus non-DNA based disease risk estimates versus only non-DNA based disease risk estimates; or disease risk estimates based on DNA versus no disease risk estimates.

The primary outcome was performance of a behaviour that could reduce the risk of disease. Behaviours included smoking, alcohol consumption, diet, and physical activity. We only included studies that measured at least one of the primary outcomes. Secondary outcomes were motivation to change behaviour and levels of depression and anxiety.

### Data extraction and synthesis

Two authors prescreened all search results (titles and abstracts) against the inclusion criteria. Studies selected by either or both authors were subjected to a full text assessment. Two authors independently assessed the selected full text articles for inclusion. Two authors independently extracted data on study participants, study design, interventions, outcome measures, results, and risk of bias characteristics. One author entered extracted data into Review Manager software, and these were checked by a second author. We contacted study authors for additional information about included studies as required.

Studies were analysed by type of behaviour, with data across diseases and interventions combined. We summarised study effect sizes for each outcome using forest plots. Effect sizes for dichotomous data were odds ratios, with values greater than one favouring the intervention group. Effect sizes for continuous outcomes were standardised mean differences, centred on zero, with values greater than zero favouring the intervention group and those less than zero favouring the comparison group. When different studies reported either dichotomous or continuous data for the same outcome, we combined these data using the generic inverse variance method, and we reported effect sizes as standardised mean differences. This involved following the methods outlined in the Cochrane handbook (sections 7.7.7. and 9.4.6)^26^: computing standard errors for these studies by entering the data separately as dichotomous and continuous outcome type data, as appropriate, and converting the confidence intervals for the resulting log odds ratios and standardised mean differences into standard errors. Log odds ratios were then converted to standardised mean differences by multiplying each by the required constant. We obtained pooled effect sizes with 95% confidence intervals using a random effects model applied on the scale of standardised mean differences and log odds ratios. We tested for heterogeneity using the χ^2^ test and quantified it using the I^2^ statistic, with a value of 50% or greater considered to represent substantial heterogeneity.^26^

If multiple indices of a given behavioural outcome were reported, we used the most stringent and valid measure of behaviour available (eg, an objective measure such as biochemically validated smoking cessation). When a study had more than one follow-up time point, we used data from the longest follow-up available. Final values were always used rather than changes from baseline. When there were multiple intervention and control arms, we chose to compare with that which allowed the purest isolation of the effect of the DNA risk communication component.

### Subgroup analysis

When data were available, we examined the effect of a genetic test result within those participants receiving DNA based disease risk estimates, comparing the effect of communicating the presence versus the absence of a risk-conferring genotype (in this context, a variant associated with an increased likelihood of disease).

### Treatment of missing data

We analysed data according to participants’ randomised groups, accounting for missing data where possible, using data as provided by authors or, for dichotomous outcomes when data were not provided, assuming that participants with missing outcomes were engaging in the risk increasing behaviour (eg, continuing to smoke). When such analysis was not possible (due to missing data or outcomes reported as continuous data) owing to the problematic nature of imputation without available individual level data, we analysed outcomes as reported.

### Assessments of risk of bias and quality of evidence

We assessed the methodological characteristics of included studies in accordance with Cochrane guidance,^26^ including assessment of sequence generation, allocation concealment, blinding, incomplete outcome data, selective outcome reporting, and other bias. For each criterion, we determined whether this represented a low, unclear, or high risk of bias, and based on the individual domains we generated a summary risk of bias assessment. If the judgment in at least one domain was “high risk of bias” then we determined the summary risk of bias to be high. We judged the summary risk of bias to be low only if judgments in all domains were “low risk of bias.” The summary risk of bias contributed to the GRADE assessment of the quality of evidence, which was applied to each primary outcome in terms of the extent of our confidence in the estimates of effects. GRADE criteria for assessing quality of evidence encompass study limitations, inconsistency, imprecision, indirectness, publication bias, and other considerations.^27^

### Patient involvement

No patients were involved in setting the research question or the outcome measures, nor were they involved in developing plans for design or implementation of the study. No patients were asked to advise on interpretation or writing up of results. There are no plans to disseminate the results of the research to study participants or the relevant patient community.

## Results

Overall, 10 515 identified references were screened for possible inclusion. Eighteen studies met the inclusion criteria. Figure 1 outlines the search and screening process and table 1 gives details of the included studies. Studies were excluded for several reasons: ineligible study design, not including a relevant outcome measure, no personalised DNA based disease risk estimates, no eligible comparison, and an ongoing study yet to report its results.

**Figure f1:**
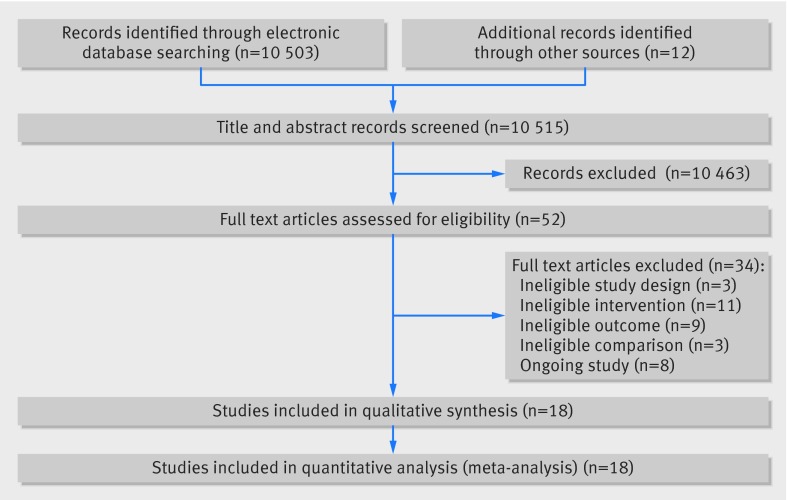
**Fig 1** Search and screening process

**Table 1 tbl1:** Characteristics of included studies

Study	Country	Setting and participants	Study design	Intervention	Comparison	Outcome(s) selected for review	Timing of outcome assessment
Audrain et al, 1997^28^^, 29^	USA	Smokers in stop smoking clinic	Randomised controlled trial	DNA based+non-DNA based disease risk estimates (CYP2D6 genotype status+carbon monoxide level readings+smoking cessation consultation). Disease risk: lung cancer	Non-DNA based disease risk estimates (carbon monoxide level readings+smoking cessation consultation)	Self reported smoking cessation (abstinence over previous 30 days)	2 months, 12 months
Chao et al, 2008^30^	USA	Individuals with family history of Alzheimer’s disease recruited from community	Randomised controlled trial	DNA based+non-DNA based disease risk estimates (education session+APOE genotype (e4+ or e4-)+individualised lifetime risk estimate). Disease risk: Alzheimer’s disease	Non-DNA based disease risk estimates (education session+individualised numerical risk estimate based on family history and sex)	Self reported health behaviour change (dietary, exercise, medicationvitamin use)	12 months
Glanz et al, 2013^31^	USA	Individuals with family history of melanoma recruited from outpatient clinic	Cluster randomised controlled trial*	DNA based disease risk estimates (CDKN2A and MC1R genotyping, genetic counselling on associated risks, and skin cancer prevention brochure). Disease risk: melanoma	No disease risk estimates (usual care of skin cancer prevention brochure)	Self reported sun protection behaviours (sun protection habits index)	4 months
Godino et al, unpublished^32^ (trial data obtained from authors)	UK	General population recruited from ongoing population based study	Randomised controlled trial	DNA based disease risk estimates (standard lifestyle advice plus genetic risk estimate for type 2 diabetes, including residual lifetime risk estimate by age and sex). Disease risk: type 2 diabetes	Non-DNA based disease risk estimates (standard lifestyle advice plus phenotypic risk estimate for type 2 diabetes (based on, for example, family history, anthropometric measures))	Physical activity assessed objectively with monitor. Self reported fruit and vegetable consumption	8 weeks
Grant et al, 2013^33^	USA	Overweight individuals at increased diabetes risk, primary care setting	Randomised controlled trial	DNA-based disease risk estimates (genetic risk feedback (summing 36 single nucleotide polymorphisms associated with type 2 diabetes) placing genetic risk within context of overall diabetes risk, 12 week diabetes prevention programme). Disease risk: type 2 diabetes	No disease risk estimates (untested controls also participated in 12 week diabetes prevention programme)	Behavioural support programme attendance	12 weeks
Hendershot et al, 2010^34^	USA	Individuals participating in study of drinking behaviour	Randomised controlled trial	DNA based disease risk estimates (web-based genetic feedback of genotype associated with alcohol-related cancer risk). Disease risk: alcohol-related cancers	No disease risk estimates (web based attention-control feedback of generic information)	Self reported frequency of alcohol use	30 days
Hieteranta-Luoma et al, 2014^35^	Finland	Healthy individuals from general population	Randomised controlled trial	DNA based disease risk estimates (apoE genotype status with personalised feedback plus additional voluntary medical consultations). Disease risk: cardiovascular disease	No disease risk estimates (no feedback of apoE genotyping, general health information on lifestyle and cardiovascular disease risk)	Self reported consumption of fruit and vegetables, alcohol consumption, and physical activity	10 weeks, 6 months, 12 months
Hishida et al, 2010^36^	Japan	Smokers in workplace	Quasi-randomised controlled trial	DNA based disease risk estimates (L-myc EcoRI polymorphism status). Disease risk: lung or oesophageal cancer	No disease risk estimates (no intervention)	Self reported smoking status (quit smoking)	12 months
Hollands et al, 2012^37^^, 38^	UK	First degree relatives of individuals with Crohn’s disease	Cluster randomised controlled trial*	DNA based disease risk estimates (genetic risk estimate for developing Crohn’s disease (also based on familial risk and smoking status)+risk assessment booklet by post and brief smoking cessation advice by telephone). Disease risk: Crohn’s disease	Non-DNA based disease risk estimates (phenotypic risk estimate for developing Crohn’s disease+risk assessment booklet by post and brief smoking cessation advice by telephone)	Biochemically validated smoking cessation	6 months
Ito et al, 2006^39^	Japan	Outpatient smokers in cancer centre hospital	Quasi-randomised controlled trial	DNA based disease risk estimates (information session+L-myc EcoRI polymorphism status+follow-up posted checklist). Disease risk: lung or oesophageal cancer	No disease risk estimates (no intervention)	Self reported smoking status	3 months, 9 months
Komiya et al, 2006^40^	Japan	Employees of a manufacturing factory	Randomised controlled trial	DNA based disease risk estimates (ALDH2 genotype plus information on associated disease risk from alcohol). Disease risk: cancers	No disease risk estimates (intervention received at later date)	Self reported weekly alcohol intake	18 months
Marteau et al, 2004^41^	UK	Adults attending lipid clinics for assessment	Randomised controlled trial	DNA based+non-DNA based disease risk estimates (routine clinical diagnosis of familial hypercholesterolaemia+cholesterol results+LDLR mutation status feedback+lifestyle advice). Disease risk: familial hypercholesterolaemia	Non-DNA based disease risk estimates (routine clinical diagnosis of familial hypercholesterolaemia+cholesterol results+lifestyle advice)	Self reported risk-reducing behaviour change (low fat diet, increased physical activity)	6 months
McBride et al, 2002^42^	USA	Smokers attending community health clinic	Randomised controlled trial	DNA based disease risk estimates (feedback of GSTM1 status in booklet with advice on smoking risks+4 telephone counselling sessions over follow-up period). Disease risk: lung cancer	No disease risk estimates (quit advice+referral to smoking specialist+quit guide and nicotine patches where required)	Self reported smoking abstinence in past 7 days	6 months, 12 months
Meisel et al, 2015^43^^, 44^	UK	Students recruited from a university	Randomised controlled trial	DNA based disease risk estimates (obesity gene (FTO) feedback, plus weight control advice leaflet). Disease risk: obesity	No disease risk estimates (weight control advice leaflet, genetic feedback given at later date)	Self reported risk-reducing diet and physical activity behaviours	1 month
Nielsen et al, 2014^45^	Canada	Online recruitment of healthy individuals	Randomised controlled trial	DNA based disease risk estimates (genetic tests for caffeine metabolism, vitamin C utilisation, sweet taste perception, and sodium sensitivity (angiotensin converting enzyme) linked to disease risk, with personalised results and dietary recommendations). Disease risk: sodium sensitive hypertension	No disease risk estimates (dietary recommendations based on current guidelines; genetic feedback was given at later date)	Self reported sodium intake	3 months, 12 months
Sanderson et al, 2008^46^	UK	Smokers in stop smoking clinic	Randomised controlled trial	DNA based disease risk estimates (leaflet+20 minute quit smoking intervention+GSTM1 status feedback). Disease risk: lung cancer	No disease risk estimates (leaflet+20 minute quit smoking intervention)	Self reported smoking status (quit smoking)	1 week, 2 months
Voils et al, 2015^47^^, 48^	USA	Overweight/obese veteran outpatients, primary care setting	Randomised controlled trial	DNA based disease risk estimates+non-DNA based disease risk estimates (genetic testing for diabetes related genes with personalised feedback+conventional diabetes risk counselling and brief lifestyle counselling). Disease risk: type 2 diabetes	Non-DNA based disease risk estimates (education on age related macular degeneration, cataracts, glaucoma+conventional diabetes risk counselling and brief lifestyle counselling)	Self reported dietary energy intake and physical activity	3 months, 6 months
Weinberg et al, 2014^49^^, 50^	USA	Individuals with average risk status for colorectal cancer who did not adhere to screening recommendations, primary care setting	Randomised controlled trial	DNA based disease risk estimates (feedback on combination of MTHFR polymorphisms and serum folate levels, and risk counselling). Disease risk: colorectal cancer	No disease risk estimates (usual care with no risk counselling)	Colorectal screening assessed by manual and electronic medical chart review	3 weeks, 6 months

*Family clusters were randomised but treated as individual randomisation because reported that clustering did not affect results.

The studies were principally carried out in outpatient or primary care clinics or various community populations. Five studies communicated the genetic risks for lung or oesophageal cancer to smokers^28^ ^36^ ^39^ ^42^ ^46^ and one study communicated the risks of Crohn’s disease to smokers.^37^ Two studies communicated the risks of oesophageal and other cancers with alcohol consumption.^34^ ^40^ One study communicated the risks of melanoma.^31^ One study communicated the risks of colorectal cancer.^49^ Three studies communicated the risk of type 2 diabetes.^32^ ^33^ ^47^ Three studies communicated the risks of heart disease, cardiovascular disease, or hypertension.^35^ ^41^ ^45^ One study communicated predictive genetic testing for Alzheimer’s disease.^30^ One study communicated the genetic risks of obesity.^43^ Eight studies were conducted in the USA,^28^ ^30^ ^31^ ^33^ ^34^ ^42^ ^47^ ^49^ five in the UK,^32^ ^37^ ^41^ ^43^ ^46^ three in Japan,^36^ ^39^ ^40^ and one study was conducted in each of Finland^35^ and Canada.^45^ The mean ages of participants, where reported, ranged from 30 to 56 years, and the sex mix of participants ranged between 0% and 73% female.

### Primary outcome analysis

In separate forest plots we show the results for dichotomous outcome data only (fig 2), continuous outcome data only (fig 3), and combined dichotomous and continuous outcome data (fig 4).

**Figure f2:**
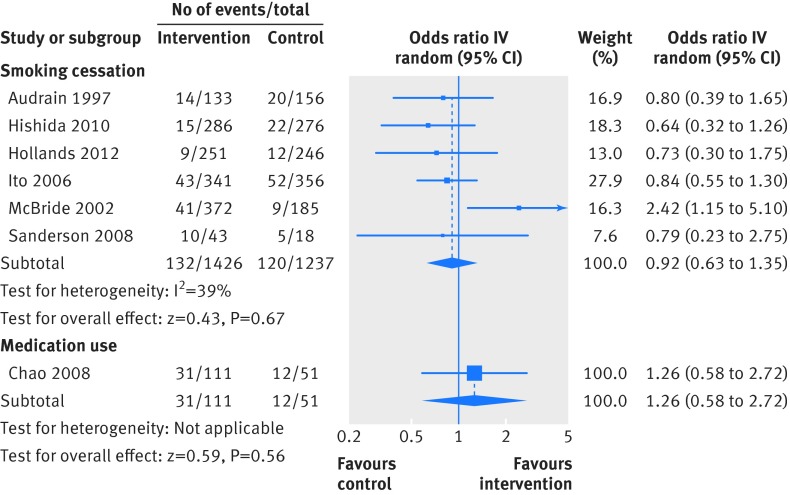
**Fig 2** Primary outcome analysis: smoking cessation; medication use

**Figure f3:**
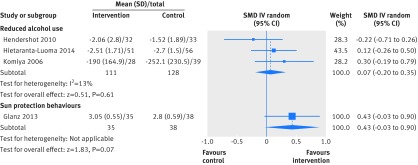
**Fig 3** Primary outcome analysis: reduced alcohol use; sun protection behaviours. SMD=standardised mean difference

**Figure f4:**
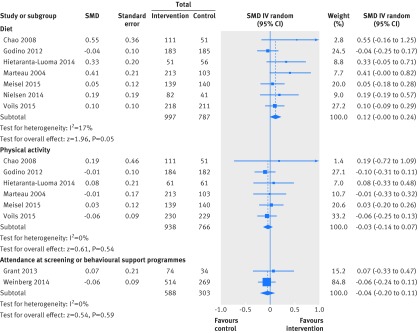
**Fig 4** Primary outcome analysis: diet; physical activity; attendance at screening or behavioural support programmes. SMD=standardised mean difference

#### Smoking cessation

Six studies assessed smoking cessation,^28^ ^36^ ^37^ ^39^ ^42^ ^46^ all but one^37^ using self report measures. The genetic risks communicated were for lung or oesophageal cancer^28^ ^36^ ^39^ ^42^ ^46^ and Crohn’s disease.^37^ Comparisons were between DNA based risk estimates versus no risk estimates for four of six studies,^36^ ^39^ ^42^ ^46^ with one study comparing DNA based plus non-DNA based risk estimates versus only non-DNA based risk estimates,^28^ and one study comparing DNA based versus non-DNA based risk estimates.^37^ Pooled analysis (n=2663) showed no significant effect of DNA based risk communication on smoking cessation (odds ratio 0.92, 95% confidence interval 0.63 to 1.35, P=0.67; I^2^=39%, fig 2). Within intervention arm subgroup analysis, assessing the effect of the presence (versus absence) of a risk-conferring genotype, was possible for five of the six studies.^36^ ^37^ ^39^ ^42^ ^46^ Pooling these data revealed no evidence of a benefit from communicating the presence of a risk-conferring genotype (odds ratio 1.26, 95% confidence interval 0.81 to 1.97, P=0.30).

#### Medication use

One study (n=162) communicated the genetic risk of Alzheimer’s disease and assessed self reported medication use to reduce this risk, at 12 month follow-up.^30^ The comparison was between DNA based plus non-DNA based risk estimates versus only non-DNA based risk estimates. The odds ratio of 1.26 (95% confidence interval 0.58 to 2.72, P=0.56) suggested no effect of DNA based risk communication (fig 2). In subgroup analysis comparing those receiving a positive versus a negative APOE e4 disclosure, the odds ratio was 2.61 (95% confidence interval 1.09 to 6.23, P=0.03), indicating a positive effect on medication use of information concerning the presence of a risk-conferring genotype.

#### Alcohol use

Three studies^34^ ^35^ ^40^ assessed self reported alcohol use, with genetic risks communicated for cancers^34^ ^40^ and for cardiovascular disease.^35^ Comparisons were between DNA based risk estimates versus no risk estimates. Pooled data (n=239) revealed no evidence of an effect of DNA based risk communication on reducing alcohol use (standardised mean difference 0.07, 95% confidence interval −0.20 to 0.35, P=0.61, I^2^=13%, fig 3). Subgroup analysis of data from one study,^35^ showed no effect of communicating a high risk genotype (standardised mean difference 0.17, 95% confidence interval −0.42 to 0.76, P=0.57).

#### Sun protection behaviours

One study (n=73) communicated the risk of melanoma and assessed self reported sun protection behaviours.^31^ The comparison was between DNA based risk estimates versus no risk estimates. The standardised mean difference was 0.43 (95% confidence interval −0.03 to 0.90, P=0.07), suggesting no effect of DNA based risk communication (fig 3). Subgroup analysis was not possible.

#### Diet

Seven studies assessed self reported dietary behaviour.^30^ ^32^ ^35^ ^41^ ^43^ ^45^ ^47^ The genetic risks communicated were for type 2 diabetes,^32^ ^47^ obesity,^43^ familial hypercholesterolaemia,^41^ Alzheimer’s disease,^30^ cardiovascular disease,^35^ and hypertension.^45^ Comparisons were between DNA based risk estimates versus no risk estimates for three studies,^35^ ^43^ ^45^ with three studies comparing DNA based plus non-DNA risk estimates versus only non-DNA based risk estimates,^30^ ^41^ ^47^ and one study comparing DNA based risk estimates versus non-DNA based risk estimates.^32^ Pooled data from these studies (n=1784) showed no significant evidence of a benefit from DNA based risk communication (standardised mean difference 0.12, 95% confidence interval −0.00 to 0.24, P=0.05, I^2=^17%, fig 4). Pooled subgroup analysis of data from three studies,^30^ ^35^ ^45^ showed no effect of communicating a high risk genotype (standardised mean difference 0.18, 95% confidence interval −0.13 to 0.50, P=0.25).

#### Physical activity

Six studies assessed physical activity as an endpoint behaviour,^30^ ^32^ ^35^ ^41^ ^43^ ^47^ all but one^32^ using self report measures. The genetic risks communicated were for type 2 diabetes,^32^ ^47^ obesity,^43^ familial hypercholesterolaemia,^41^ Alzheimer’s disease,^30^ and cardiovascular disease.^35^ Comparisons were between DNA based risk estimates versus no risk estimates for two studies,^35^ ^43^ with three studies comparing DNA based plus non-DNA based risk estimates versus only non-DNA based risk estimates,^30^ ^41^ ^47^ and one study comparing DNA based versus non-DNA based risk estimates.^32^ Pooled data from these studies (n=1704) revealed no evidence of an effect of DNA based risk communication (standardised mean difference −0.03, 95% confidence interval −0.14 to 0.07, P=0.54, I^2^=0%, fig 4). Pooled subgroup analysis of data from two studies^30^ ^35^ showed no effect of communicating a high risk genotype (odds ratio 1.23, 95% confidence interval 0.49 to 3.11, P=0.65).

#### Attendance at screening or behavioural support programmes

Two studies assessed attendance at screening or behavioural support programmes^33^ ^49^ following communication of genetic risks for type 2 diabetes^33^ and colorectal cancer.^49^ Comparisons were between DNA based risk estimates versus no risk estimates. Pooled analysis (n=891) suggested no effect of DNA based risk communication (standardised mean difference −0.04, 95% confidence interval −0.20 to 0.11, P=0.59, I^2^=0%, fig 4). It was possible to conduct subgroup analysis with data from both studies, which showed no effect of communicating a high risk genotype (standardised mean difference −0.16, 95% confidence interval −0.47 to 0.16, P=0.33).

### Secondary outcomes

The few data reported on prespecified secondary outcomes of motivation to change behaviour and of depression and anxiety provided no evidence of any intervention impact on these outcomes. Five studies assessed motivation or intention to change behaviour,^32^ ^33^ ^34^ ^36^ ^46^ two studies measured depression,^41^ ^46^ and three studies measured anxiety.^32^ ^41^ ^46^ In all cases, confidence intervals included no effect.

### Assessment of risk of bias and quality of evidence

Only four of the 18 studies were considered to have a low summary risk of bias, having met all of the specified criteria (fig 5).^32^ ^33^ ^37^ ^49^ The inability of 14 of 18 studies to meet criteria for low summary risk of bias reflected both a lack of clarity in reporting and a failure or inability to safeguard against risk of bias. In terms of GRADE assessment of the quality of the evidence across outcomes, evidence was determined to be of low quality for all outcomes other than attendance at screening or behavioural support, meaning limited confidence is placed in the effect estimates. Evidence was downgraded twice for these outcomes owing to study limitations (with all or most information for the outcome from studies at high or unclear risk of bias) and imprecision (with sample sizes failing to meet the optimal information size and/or 95% confidence intervals for the summary effect estimate overlapping no effect and including appreciable benefit or harm). For the outcome of attendance at screening or behavioural support, the evidence was downgraded only once owing to imprecision (and not study limitations, as information came from studies at low risk of bias). Therefore, the evidence for this outcome was assessed to be of moderate quality.

**Figure f5:**
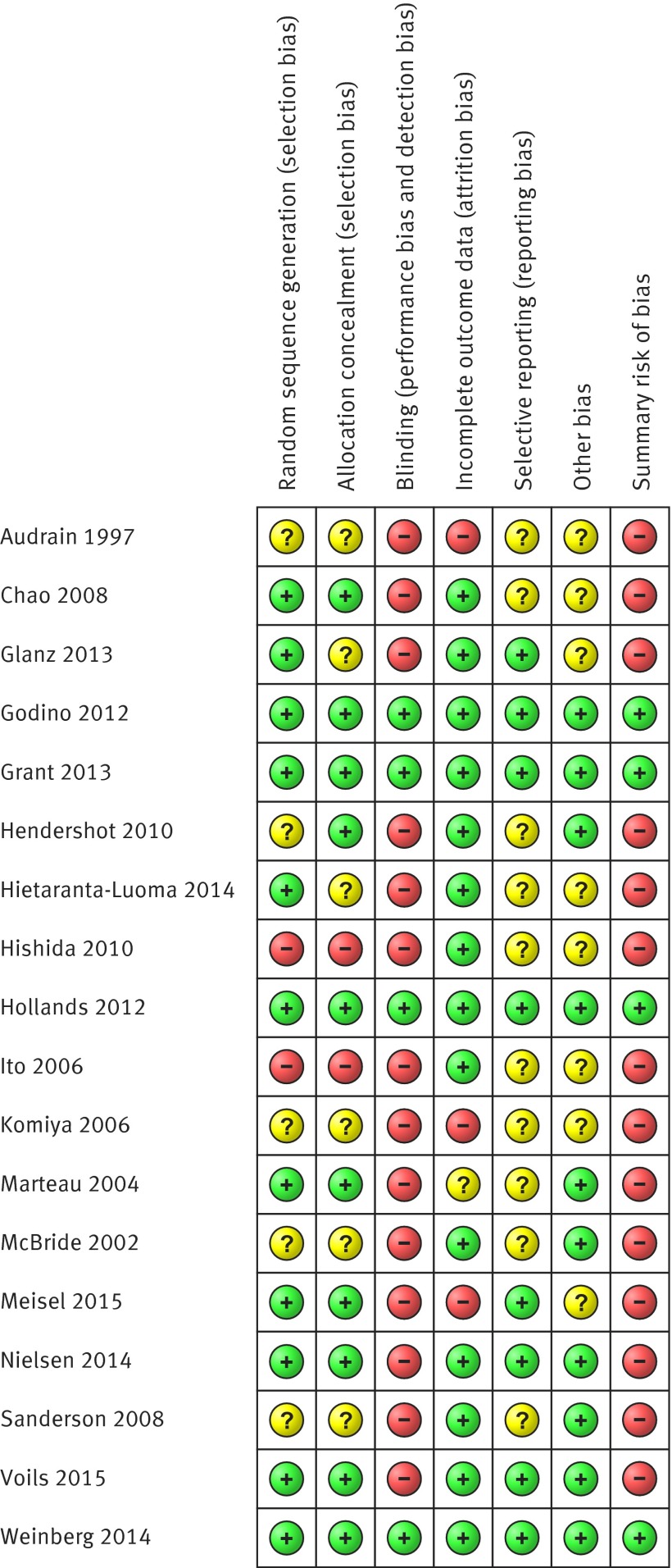
**Fig 5** Assessment of risk of bias

## Discussion

The evidence in this review suggests that communicating DNA based disease risk estimates has little or no effect on health related behaviour. The evidence for concluding an absence of effect was strongest for smoking cessation and physical activity, where for both, six studies contributed comparably consistent effects, with pooled point estimates of effect size close to unity, supported by relatively narrow 95% confidence intervals. The evidence concerning attendance at screening or behavioural support shared similar characteristics and indicated an absence of effect, although findings were based on only two studies (albeit both well conducted trials). The results from the seven studies on dietary behaviour are compatible with a small effect of genetic risk communication and with a narrow pooled confidence interval. For all other behaviours, data were considerably fewer. There were also no effects on motivation to change behaviour, and no adverse effects on depression or anxiety, although again there were few data for these secondary outcomes. Finally, the supplementary subgroup analyses within participants in the intervention arms only, suggest that there is no clear effect of genetic test result. Only one of six analyses showed a statistically significant effect of communicating the presence versus absence of a risk conferring mutation, and this was derived from one study.

### Strengths and weaknesses of this review

We conducted the review using rigorous Cochrane methods to minimise the risk of bias. We included quantitative synthesis using meta-analysis and systematic assessment of risk of bias of included studies and of quality of the evidence by outcome, and we identified a substantive body of randomised studies able to inform our specified aims. Previous reviews had identified few clinical studies using randomised designs, did not include quantitative syntheses of effects on behaviour, or were focused on single behaviours.

However, our review does have several limitations, linked to limitations of the available evidence. Principally, we found that several studies were limited in their ability to address the review objective. They were often underpowered to detect plausible small effects of risk information on behaviour, and many of the studies (10 of 18) were judged to have control groups of low relevance because their content differed from the intervention group in more than only the absence of DNA based information on disease risk. For example, one study that produced a medium sized effect on behaviour had an intervention group that differed from the control group both in the use of DNA based risk communication and in the provision of telephone counselling.^42^ Also, few included studies were determined to be at low summary risk of bias. In particular, the failure or inability to use valid measures of behaviour may have introduced error and bias. While we acknowledge that the use of self report measures is sometimes necessary, included studies typically used self report measures even when viable objective measures were available (for example, in relation to smoking cessation).^51^ Participants and providers are not blinded to the intervention and it is important that outcome assessors are blinded, but this was rarely the case (at least as reported), and, where self report measures are used, is not possible. The potential for selective outcome reporting was also notable, with few instances of trial registration or published protocols. The substantive risk of bias and seemingly poor quality of many of the included studies, and the relative imprecision of the effect estimates, suggests caution in interpreting the results.

### Interpretation of study results

We outlined three possible competing hypotheses on the possible behavioural impact of DNA based disease risk information evident in the literature—that it strongly motivates risk-reducing behaviour change, that it demotivates risk-reducing behaviour change, and, finally that, at best, it has only a small effect on risk-reducing behaviour. Our results do not support the first two hypotheses, but are consistent with the third, suggesting that high expectations of the potency of such communications to change behaviour are unfounded. This is consistent with the results of a recent cohort study reporting no impact on diet or physical activity of direct-to-consumer genome-wide testing.^52^ It is also in accord with the results of a Cochrane review in which the authors concluded that the current evidence does not support the hypothesis that biomedical risk assessment increases smoking cessation.^14^ The theoretically oriented literature on behaviour change also highlights the typically small effect of risk communication on behaviour.^12^ While the results of the current review are strongly suggestive of, at most, small effects on health behaviours, high quality research evidence is currently insufficient to engender confidence of this for each individual behaviour included in the review. However, given the overall pattern of the combined evidence, any additional large scale trials, even if better designed and conducted, need a clear justification. Such justification would be based on incrementally developed evidence indicating that efficacy of a clinically important degree is possible (that is, higher than the priors based on this review) given the particular characteristics of the intervention and target population.

Previous reviews of the behavioural impact of genetic risk communication have included non-randomised studies, predominantly of those with family histories of breast, ovarian, and colorectal cancer, with the dominant behaviours reported being screening or prophylactic surgery. These indicate an increase in screening and prophylactic surgery, particularly among those found to be carriers—that is, those with an increased risk of disease.^16^ ^17^ ^18^ ^19^ Such findings suggest that DNA based risk assessments are more likely to motivate clinical means of reducing risk (such as undergoing surgery or attending screening) than behavioural means (such as altering smoking, diet, or physical activity behaviours) that are the main focus of this review.^53^ In spite of this, the one large and well conducted trial included in this review^49^ that assessed the impact of DNA risk communication on colorectal screening found no effect on uptake.

### Implications for public health and research

The available evidence does not provide support for the expectations raised by researchers and proponents of personalised medicine as well as direct-to-consumer testing companies that the receipt of results from DNA based tests for gene variants that confer increased risk of common complex diseases motivates behaviour change. Concerns that communicating DNA based disease risk estimates may demotivate behaviour change are also unsupported by the results of this review. Where such tests exist, be it in public or private sector domains, their use warrants the collection of evidence on behaviour change as part of research protocols, thereby contributing to the limited existing evidence base. At present there is little evidence to suggest that simply communicating the results of DNA tests has a role in strategies aimed at improving population health by motivating risk-reducing behaviour change.^54^ Such tests may, however, have a role in such strategies if supplemented by the offer of effective behaviour change interventions. DNA testing, alone or in combination with other assessments of disease risk, may have a role in stratifying populations by risk, to enable clinical and behavioural interventions—such as screening tests, surgery, and drug treatments—to be targeted at those at increased risk.^55^

The communication of genetic information may differ in respect to how much it is framed as a “risk” to health, or used to inform recommendations for wellness (even if these are derived from associations with increased risk). For example, nutrigenomic information may not be presented or characterised as risk information but may be used to inform behavioural recommendations, which can be highly specific and targeted. This is demonstrated by one of the included studies,^45^ which used nutrigenomic testing to provide specific intake recommendations for foods. However, as yet there are too few trials to assess whether this type of genetic testing has a different impact from more traditional genetic testing providing information about the likelihood of a health harm.

Given the continued high expectations for the communication of DNA based disease risk estimates to motivate risk-reducing behaviour change, it is important that any additional randomised controlled trials are conducted using methodologically robust designs. These would be powered to detect possible small effects on behaviour (that might have important population consequences), and conducted and reported cognisant of the risks of bias—for example, by incorporating prespecified outcomes, valid measures of behaviour, and the blinding of outcome assessors.

### Conclusion

The results of this review suggest that communicating DNA based disease risk estimates has little or no effect on risk-reducing health behaviour. Existing evidence does not support expectations that such interventions could play a major role in motivating behaviour change to improve population health.

**What is already known on this topic**

Genetic testing is being increasingly used in a growing number of healthcare settings and in direct-to-consumer testing for a range of common complex disorders
There is an expectation that communicating DNA based disease risk estimates will motivate changes in key health behaviours, including smoking, diet, and physical activity
There is a need for a rigorous systematic review to examine whether communicating genetic risks does indeed motivate risk-reducing behaviour change

**What this study adds**

The results of this updated systematic review with meta-analysis using Cochrane methods suggest that communicating DNA based disease risk estimates has little or no impact on risk-reducing health behaviour
Existing evidence does not support expectations that such interventions could play a major role in motivating behaviour change to improve population health

## Web Extra

Extra material supplied by the author

**Web appendix:** Medline search strategy

## Notes

We thank Barb Biesecker and Hilary Burton for their helpful comments on a draft of this paper.

Ethical approval: Not required.

Data sharing: All data used for the review are available from the authors.

Transparency: The manuscript’s guarantor (TMM) had full access to all of the data in the review and takes responsibility for the integrity of the data and accuracy of the data analysis. She accepts full responsibility for the conduct of the review and has controlled the decision to publish. She affirms that the manuscript is an honest, accurate, and transparent account of the study being reported; that no important aspects of the study have been omitted; and that any discrepancies from the study as planned (and, if relevant, registered) have been explained.
