# Supplementary material for: The impact of communicating genetic risks of disease on risk-reducing health behaviour: systematic review with meta-analysis
Source: BMJ. 2016 Mar 15;352:i1102. doi: 10.1136/bmj.i1102 (PMC4793156; doi:10.1136/bmj.i1102)
Supplement: Supplementary file 1 — Web appendix: Medline search strategy [file holg030916.ww1_default.pdf]

Appendix – MEDLINE search

MEDLINE

1. genetic services/
2. genetic testing/
3. genetic counseling/
4. genetic predisposition to disease/
5. or/1-4
6. ((gene or genes or genetic* or genotype*) adj3 (test* or assess* or risk* or susceptib* or predispos* or disease* or screen* or prognos* or predict* or servic*)).ti,ab,kw.
7. counseling/ or directive counseling/
8. health communication/
9. (consult* or assess* or support* or inform* or advis* or advice or counsel* or educat* or shar* or communicat* or teach* or discuss* or decid* or decision*).ti,ab,kw.
10. patient education as topic/
11. or/7-10
12. 6 and 11
13. 5 or 12
14. (adher* or nonadher* or complian* or noncomplian*).ti,ab,kw.
15. patient compliance/
16. exp health behavior/
17. risk reduction behavior/
18. (diet* or smok* or tobacco or nicotine or alcohol or weight or activ* or behavio* or attitud* or motivat* or intention* or perceiv* or perception* or decid* or decision*).ti,ab,kw.
19. or/14-18
20. 13 and 19
21. randomized controlled trial.pt.
22. controlled clinical trial.pt.
23. randomized.ab.
24. placebo.ab.
25. drug therapy.fs.
26. randomly.ab.
27. trial.ab.
28. groups.ab.
29. or/21-28
30. exp animals/ not humans.sh.
31. 29 not 30
32. 20 and 31
33. 32 and (2010* or 2011* or 2012* or 2013* or 2014* or 2015*).ed,ep,dc.
